# Supplementary material for: EnzML: multi-label prediction of enzyme classes using InterPro signatures
Source: BMC Bioinformatics. 2012 Apr 25;13:61. doi: 10.1186/1471-2105-13-61 (PMC3483700; doi:10.1186/1471-2105-13-61)
Supplement: Addtional file 5 — The Java code to format the data files, evaluate and predict. The file enzml_java_code.tar.gz contains the Java code used to format database data to ARFF and XML formats, to execute cross and train-test (jackknife) evaluations and to record evaluation results to database. More information is included in the readme.txt file and the Javadoc files. The code can be used with a MySQL database. To use a different database software, other JDBC drivers might be required. [file 1471-2105-13-61-S5.gz › java_code/utils/doc/uk/ac/ed/inf/utils/stats/UniformRandomUtils.html]

UniformRandomUtils


---


|  |  |  |
| --- | --- | --- |
| **Overview** \| **Package** \| **Class** \| **Use** \| **Tree** \| **Deprecated** \| **Index** \| **Help** | |  |
| **PREV CLASS**   NEXT CLASS | **FRAMES**    **NO FRAMES**     **All Classes** |  |
| SUMMARY: NESTED \| FIELD \| CONSTR \| METHOD | DETAIL: FIELD \| CONSTR \| METHOD |  |


---


## uk.ac.ed.inf.utils.stats Class UniformRandomUtils

```
java.lang.Object
  uk.ac.ed.inf.utils.stats.UniformRandomUtils
```

---

``` public class UniformRandomUtils extends java.lang.Object ```

Utilities for extracting with uniform random distribution

**Version:**
:   29 Jun 2008

**Author:**
:   Luna De Ferrari luna.deferrari-at-ed.ac.uk

---

**Constructor Summary**

`UniformRandomUtils()`


| **Method Summary** | |
| --- | --- |
| `static int` | `extractInteger(int size)`             Returns a random integer between 0 and (size-1) |
| `static java.util.Map.Entry` | `extractUniformRandomMapEntry(java.util.Map map)`             Extracts a random entry (key-value association) from a map |
| `static java.util.Map` | `getUniformRandomSubmap(java.util.Map map, double percentage)`             Returns a submap of randomly picked map entries |

**Methods inherited from class java.lang.Object**

`equals, getClass, hashCode, notify, notifyAll, toString, wait, wait, wait`

**Constructor Detail**

### UniformRandomUtils

```
public UniformRandomUtils()
```


**Method Detail**

### extractInteger

```
public static int extractInteger(int size)
```

:   Returns a random integer between 0 and (size-1)

    :   **Parameters:**: `size` - size-1 is the biggest integer that can be extracted **Returns:**: a random integer between 0 and (size-1)

---


### extractUniformRandomMapEntry

```
public static java.util.Map.Entry extractUniformRandomMapEntry(java.util.Map map)
```

:   Extracts a random entry (key-value association) from a map

    :   **Parameters:**: `map` - a map **Returns:**: a random (uniform probability) entry from the map

---


### getUniformRandomSubmap

```
public static java.util.Map getUniformRandomSubmap(java.util.Map map,
                                                   double percentage)
```

:   Returns a submap of randomly picked map entries

    :   **Parameters:**: `map` - the original map: `percentage` - the percentage of the original map to be put in the submap **Returns:**: the random submap


---


|  |  |  |
| --- | --- | --- |
| **Overview** \| **Package** \| **Class** \| **Use** \| **Tree** \| **Deprecated** \| **Index** \| **Help** | |  |
| **PREV CLASS**   NEXT CLASS | **FRAMES**    **NO FRAMES**     **All Classes** |  |
| SUMMARY: NESTED \| FIELD \| CONSTR \| METHOD | DETAIL: FIELD \| CONSTR \| METHOD |  |


---
